# Editorial: Microbial-based solutions to reduce contaminants in foods and beverages

**DOI:** 10.3389/fmicb.2023.1192658

**Published:** 2023-05-16

**Authors:** Bavaro Simona Lucia, Álvarez-Ordóñez Avelino, Capozzi Vittorio

**Affiliations:** ^1^Institute of Sciences of Food Production, National Research Council (CNR), Grugliasco, Italy; ^2^Department of Food Hygiene and Technology, Universidad de León, León, Spain; ^3^Institute of Food Science and Technology, Universidad de León, León, Spain; ^4^Institute of Sciences of Food Production, National Research Council (CNR), Foggia, Italy

**Keywords:** microorganism, food safety, food quality, bioremediation, food and beverage

During different stages of food production, from the farm to the fork, many foods and beverages are prone to chemical and biological contaminations, improving the risks of making them unsuitable for consumption. Globally, ~14% of food produced is lost between harvest and retail, while an estimated 17% of total global food production is wasted (11% in households, 5% in food services, and 2% in retail; United Nations, 2022; https://www.un.org/en/observances/end-food-waste-day). Recently, microorganisms have been extensively used in the food chain to preserve the safety, nutritional properties, and, more generally, quality of food products. Among the different contaminants/spoilage control strategies, biopreservation has been proposed as an alternative method (Hernández et al., 2022; Kasimin et al., 2022) to extend the shelf life of foods by inoculating selected bacteria that can inhibit the growth of undesirable contaminants (Agriopoulou et al., 2020). Moreover, many studies have been conducted on the development of microbial strategies to reduce toxic by-products of microbial origin (e.g., mycotoxins and biogenic amines), chemical contaminants (e.g., polychlorinated biphenyls, dioxin-like compounds, pesticide residues, and perchlorate), or intrinsic factors, which are part of the food product itself, such as allergens.

The goal of the present Research Topic is to give an overview of the use of microbial-based biotools and their application in the food chain. Therefore, contributions were gathered from scientists working in diverse disciplines with common interests in microbiology. The objective is that bringing together seemingly different lines of research under one cover can result in a more global understanding of biopreservation/bioremediation, and perhaps draw new insight into food safety and food quality fields.

In this perspective, it was highlighted how the microorganisms and their enzymes can be used for mycotoxin and heavy metal detoxification or for inhibiting the growth of undesired microbes in various foodstuffs and feeds.

In particular, Abraham et al. provide a comprehensive review of the microbial enzymatic transformation of major mycotoxins, discussing the potential application of enzymes for mycotoxin detoxification and promoting their implementation as a successful strategy to remove these contaminants from foods/feeds.

The study by Wang et al. is focused on the molecular mechanisms underlying cadmium (Cd) tolerance in *Hypomyces chrysospermus* (*H. chrysospermus*), a fungal parasite that grows on *Boletus* species, with a strong ability to tolerate and absorb Cd. The exposure of *H. chrysospermus* to Cd stress has revealed that large amounts of differentially expressed genes were mainly involved in the translation, amino acid metabolism, transport and catabolism, carbohydrate metabolism, folding/sorting, and degradation pathways under Cd stress, providing new insights into the detoxification mechanisms of heavy metal-tolerant fungi and improved fungal bioremediation strategies. Olowe et al. investigated the use of *Trichoderma* species in the sustainable management of *Fusarium* infections, affecting oak and rice crops and characterizing its effects by biological and molecular tools. The obtained results showed that more members of the genus *Trichoderma* could have promising antagonistic prowess against fungi of economic importance, such as *Fusarium* species.

Shen et al. evaluated the potential risk of foodborne pathogen contaminations during blueberry production in the field, with or without derived ammonium sulfate (AS) fertilizer, by monitoring total coliforms and generic *Escherichia coli* and the main foodborne pathogens, including Shiga toxin-producing *Escherichia coli* (STEC), *Salmonella*, and *Listeria monocytogenes*. The study demonstrated that, under good agricultural practices, blueberries produced in fields, with or without manure-derived fertilizer AS, had food safety concerns only for *Salmonella* in early-season soil samples. On the contrary, total coliforms and generic *Escherichia coli, STEC*, and *L. monocytogenes* were all below the detection limit in all samples collected during production seasons.

Finally, the original research article by Donaghy et al. illustrated, nowadays, as big data analysis, in a dynamic risk management system, could be used in real time to identify hazards and control STEC risks related to leafy greens. At the same time, Dong and Feng highlighted that the high-throughput sequencing methods and analysis for microbiome research, combined with a farming food safety practice survey, can be used to explore the plant and environmental microbiomes in hydroponic cropping ecosystems. These outputs could provide customized and targeted improvement strategies throughout the hydroponic chain.

In conclusion, these studies present the progress in microbial bioremediation, food microbiology, and food biotechnologies, providing new information for the design of “*Microbial-based solutions to reduce contaminants in foods and beverages*.” However, deep investigations are still needed to develop precision fermentation biotools to improve the global quality/safety of food and beverage products and their economic, social, and environmental repercussions.

## Author contributions

All authors contributed to the article and approved the submitted version.
